# Twenty‐Year Trends in Colectomy Rates and Advanced Therapy Prescribing in Lothian, Scotland

**DOI:** 10.1111/apt.70240

**Published:** 2025-06-16

**Authors:** Alexander T. Elford, Nathan Constantine‐Cooke, Phil W. Jenkinson, Beatriz Gros, Nikolas Plevris, Mathew Lyons, Solomon Ong, Neil Greenlees, Victor Velasco‐Pardo, Claire O'Hare, Nicholas T. Ventham, Paul Henderson, David C. Wilson, Shahida Din, Colin L. Noble, Gareth‐Rhys Jones, Ian Arnott, Charlie W. Lees

**Affiliations:** ^1^ Edinburgh IBD Unit, Western General Hospital Edinburgh UK; ^2^ Faculty of Medicine The University of Melbourne Melbourne Australia; ^3^ MRC Human Genetics Unit Institute of Genetics and Cancer, University of Edinburgh, Western General Hospital Edinburgh UK; ^4^ Centre for Genomics and Experimental Medicine Institute of Genetics and Cancer, University of Edinburgh, Western General Hospital Edinburgh UK; ^5^ Department of Gastroenterology and Hepatology Reina Sofía University Hospital, IMIBIC, University of Córdoba Córdoba Spain; ^6^ Biomedical Research Center in Hepatic and Digestive Disease CIBEREHD Madrid Spain; ^7^ Centre for Medical Informatics Usher Institute, University of Edinburgh Edinburgh UK; ^8^ Royal Hospital for Children and Young People Edinburgh UK; ^9^ Child Life and Health, Centre for Inflammation Research University of Edinburgh Edinburgh UK; ^10^ Cancer Research UK Edinburgh Centre Institute of Genetics and Cancer, University of Edinburgh Edinburgh UK

**Keywords:** advanced therapies, biologics, colectomy, inflammatory bowel disease, ulcerative colitis

## Abstract

**Background:**

The impact of advanced therapy prescribing on colectomy rates in ulcerative colitis (UC) is unknown with conflicting published evidence.

**Aim:**

To describe advanced therapy prescribing trends and colectomy rates for patients with UC in Lothian, UK between January 1st, 2004 and December 31st, 2023.

**Methods:**

We obtained incidence and prevalence data from the Lothian IBD Registry, a rigorously validated population cohort. We report advanced therapy prescribing and colectomy data as raw numbers and annual incidence rates. We used piecewise linear regression analyses to identify temporal trends in prescription and colectomy rates.

**Results:**

The prevalence of UC increased from 216 to 441 per 100,000 population in the 20 years from 2004, culminating in a total of 4115 patients with UC in 2023. We identified 720 patients who had received an advanced therapy. Prescribing of first‐line advanced therapy increased from 0 in 2004 to 115 in 2023, equating to 0.00 and 2.82 per 100 patients with UC. We identified 563 patients of the prevalent UC population who had colectomy, of whom 68% were performed as emergencies. Absolute colectomy numbers decreased from 42 in 2004 to 7 in 2023, equating to 2.48 and 0.22 per 100 patients with UC. A join point in 2013 was found for both increased advanced therapy prescribing and decreased colectomy rates.

**Conclusion:**

The incidence of colectomy in the UC population has decreased over time while the use of advanced therapies has greatly increased.

## Graphical abstract

Prescribing of first‐line advanced therapy increased from 0 in 2004 to 115 in 2023, and absolute colectomy numbers decreased from 42 in 2004 to 7 in 2023. Piecewise linear regression found a join point in 2013 for both increased advanced therapy prescribing and decreased colectomy rates.

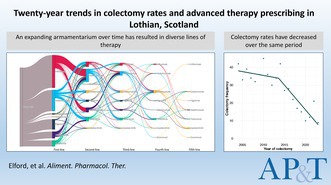

## 1 Introduction

Ulcerative colitis (UC) is a chronic inflammatory bowel disease predominantly affecting the colon, characterised by a relapsing and remitting course [1]. Conventional treatments for UC in the form of corticosteroids, thiopurines and mesalazine are often ineffective for moderate to severe cases [2]. Studies published in the pre‐biologic era found colectomy rates for UC as high as 24% (1962–1987) within 10 years of diagnosis [3, 4]. The impact of the widespread introduction of advanced therapies for UC in the past decade on colectomy rates remains unclear, with often conflicting published data on the topic [5, 6, 7, 8]. We previously published our early experience of biologic prescribing 2005–2018 inclusive for Lothian, Scotland. In this work, we noted a significant change in prescribing patterns occurred in 2015 when biologics became readily available in Scotland for treating UC [9]. We observed a decrease in colectomy rates, which correlated with the introduction and uptake of biologic therapies in UC. Since that evaluation, ustekinumab, tofacitinib, filgotinib, upadacitinib, ozanimod and etrasimod have become available in Lothian for the treatment of UC. The prescribing patterns of these new therapies, including sequencing of these therapies, and their effect on colectomy rates, have not been described.

We sought to update the Lothian data by additional data points regarding prescribing trends and colectomy rates in UC to the end of 2023. The aims of this study were to (i) describe the incidence of new advanced therapies prescriptions per year for patients with UC; (ii) describe the incidence of colectomy per year for patients with UC; and (iii) examine prescription trends for advanced therapy treatments and colectomy rates from 2004 to 2023 inclusive in Lothian.

## 2 Materials and Methods

### 2.1 Study Design

We conducted a retrospective cohort analysis of all patients with UC in Lothian over a 20‐year period from January 1st, 2004, to December 31st, 2023. Lothian's population was approaching one million people as of 2023 [10], and has one of the highest reported prevalences of IBD in the world, forecasted to affect 1% of the population by 2028 [11]. The overwhelming majority of patients with UC are treated by National Health Service (NHS) Lothian, which provides universal free healthcare. NHS Lothian includes three tertiary care hospitals (two adult and one paediatric) and one secondary care hospital. The majority of UC care occurs at the Western General Hospital for adults, which includes the Edinburgh IBD unit and Edinburgh Colorectal unit. Paediatric UC care predominantly occurs at the Royal Hospital for Children and Young People.

### 2.2 Primary and Secondary Outcomes

Our co‐primary outcomes were prescription rates of first advanced therapy for UC per year and colectomy rates per year for UC in the total prevalent population [11]. Our secondary outcomes were to describe the incidence of prescribing subsequent advanced therapies for UC and describe which treatments are used over time for various lines of therapy. We also sought to evaluate persistence rates of first‐line advanced therapies in the UC population. Our secondary outcomes were identifying the incidence of elective and emergency colectomies, evaluating early vs. late colectomy, identifying underlying indications for colectomy, identifying those who proceeded to an ileal pouch anal‐anastomosis (IPAA), describing post‐operative colectomy complications, and evaluating the trends of these over time.

### 2.3 Definitions

Patients with UC were defined based on standard clinical, endoscopic and histological criteria [12]. We used the Montreal classification to describe disease extent [13]. Advanced therapies were included if prescribed for UC and included anti‐TNF (infliximab, adalimumab and golimumab), vedolizumab, ustekinumab, JAK inhibitors (filgotinib, tofacitinib and upadacitinib) and ozanimod. Etrasimod and mirikizumab were not in use for UC in NHS Lothian during this period. We categorised infliximab use further as either acute severe ulcerative colitis (ASUC) therapy or standard therapy. We defined ASUC therapy as infliximab initially commenced as salvage therapy for a patient hospitalised with UC, and defined standard therapy as infliximab use commenced in the ambulatory care setting. We defined ASUC infliximab therapy as successful if it prevented an inpatient colectomy during that admission and considered infliximab unsuccessful if the patient required a colectomy despite salvage therapy during that admission or if the patient died due to ASUC. We censored prescribing data at (i) colectomy, (ii) death, (iii) moved services, (iv) lost to follow‐up or (v) 1st of January 2024, whichever occurred earliest. We considered patients lost to follow‐up if they had no interaction with their treating IBD service for greater than 24 months, with the censor date being the date of last documented clinical interaction. We excluded patients if they (i) received their advanced therapy for an indication other than UC, (ii) diagnosis was revised from UC to Crohn's disease (CD) or IBD‐unclassified during the study period, (iii) their advanced therapy treatment was for chronic pouchitis and (iv) patients who transiently received an advanced therapy whilst living in Lothian as a temporary resident for less than 12 months.

We classified colectomy either as a subtotal colectomy or panproctocolectomy in a UC patient for the indication of active inflammation or colorectal malignancy. We sub‐categorised colorectal malignancy as colorectal cancer, suspected colorectal cancer, high‐grade dysplasia, or low‐grade dysplasia. We defined emergency colectomy as a previously unplanned colectomy performed during a hospital admission for a UC flare, elective colectomy as a planned hospital admission for the purpose of performing a colectomy, and early colectomy within 90 days of UC diagnosis. We classified post‐operative complication severity according to the Clavien–Dindo classification [14], with grade 3–5 complications considered major complications. We further classified complications into infective versus non‐infective complications. We excluded patients who were from a different catchment area to Lothian but had a colectomy in NHS Lothian via tertiary referral, as well as patients whose colectomy histology was not consistent with UC.

### 2.4 Data Collection

We collected data by manual review of electronic health records using the TrakCare (InterSystems Corporation, Cambridge, Massachusetts, USA) system from the 1st November 2023 through to the 1st December 2024. Legacy data (typically prior to 2009) were manually uploaded either to TrakCare or SCI store, a Scottish health care information repository. We collected baseline demographic data, phenotype data, medication prescriptions, timing of advanced therapy, operation type, 30‐day post‐operative complications, and related follow‐up surgery including proctectomy and IPAA. Post‐operative complications were recorded after reviewing the inpatient notes and discharge summary for the admission of the surgery and any follow‐up presentations to hospital within 30 days of the operation date. We recorded post‐operative complications from 2009 onwards, as complication data was not readily available on the electronic medical records prior to this. For some colectomy data, 2005 rather than 2004 data is presented due to specific data for 2004 being unavailable. We identified patients using multiple clinical, administrative and research databases. We identified patients who commenced an advanced therapy from the NHS Lothian IBD Biologics Database, a prospective registry that contains biological prescriptions for IBD patients from 1st August 1999 to the present day. We also identified patients via homecare medication delivery referrals and a monthly IBD prescription referral database. Patients with colectomies were identified using the NHS Lothian pathology database, NHS Lothian Operating Room Scheduling and Office System database, TrakCare theatre lists, NHS Lothian multidisciplinary meetings database, and an inpatient coding database. We removed patient duplicates.

We obtained incidence and prevalence data from the Lothian IBD registry [11], a rigorously validated registry of the IBD population in Lothian. Actual incidence and prevalence are reported from 2008 to 2019 inclusive. We used quadratic linear modelling [15] for predicted prevalence for 2004–2007 and 2020–2023.

### 2.5 Statistical Analysis

We used SPSS Version 25 [IBM Inc., Chicago, IL, USA], Prism Version 10.0 [Graphpad Software, San Diego, CA, USA] and R 4.4.0 [R Core Team, Vienna, Austria] for statistical analyses and generation of graphs. We present descriptive statistics as medians with interquartile range [IQR] for continuous variables and frequencies with percentages for categorical variables. Sankey diagrams were used to visualise prescribing patterns. We analysed biologic prescribing and surgical rates via linear and segmental regression. The annual percentage change (APC) was computed via the estimated slopes for the covariate ‘year.’ We considered APCs not statistically significant when 95% confidence intervals crossed zero. The *p*‐value for the model selected by the regression is quoted.

### 2.6 Ethical Considerations

Our data was collected as part of clinical service evaluation under the Caldicott approval of the Lothian IBD registry (Project ID:CG/DF/CRD18002), and all data was collected as part of routine clinical care. Results describing fewer than five patients were censored to preserve confidentiality.

## 3 Results

### 3.1 Study Population

The prevalence of UC increased from 216 to 441 patients per 100,000 population between 2004 and 2023 with a total of 4115 UC patients in Lothian in 2023. We identified a total of 720 patients as having commenced an advanced therapy for UC from the 1st of January 2004 to the 31st of December 2023 (Figure S1). The median age at commencement of an advanced therapy was 39 years old (IQR 27–54) (Table 1) and the median time to first advanced therapy from diagnosis was 4.0 years (IQR 0.8–9.7) with no significant change between 2015 and 2023 (Figure S2). We identified 563 patients of the prevalent UC population who underwent a colectomy during the same time period. The median age at colectomy was 42 years old (IQR 29–56) (Table 2) whilst the median time from diagnosis to colectomy was 2.6 years (IQR 0.4–9.4) with no significant change between 2005 and 2023 (Figure S3).

**TABLE 1:** Advanced therapy cohort demographics, phenotype and therapy exposure at last follow‐up.

Demographic	All patients *n* = 720
Male sex, *n* (%)	382 (53%)
Median age at diagnosis, years, IQR	31 (22, 44)
Median age at first advanced therapy, years, IQR	39 (27, 54)
Median age of cohort, years, IQR	42 (30, 56)
Median time from diagnosis to first therapy, years, IQR	4.0 years (IQR 0.7–10.2)
Montreal Classification, *n* (%)	
E1	57 (8%)
E2	305 (42%)
E3	346 (48%)
Unknown	12 (2%)
Smoking status, *n* (%)	
Never smoker	559 (78%)
Current smoker	21 (3%)
Former smoker	134 (19%)
Unknown	6 (< 1%)
Previous colonic surgery^a^, *n* (%)	8 (1%)

Therapy exposure, *n* (%)	All patients *n* = 720
Mesalazine	651 (90%)^b^
Thiopurine	453 (63%)^c^
Infliximab	376 (52%)
Adalimumab	119 (17%)
Golimumab	15 (2%)
Vedolizumab	347 (48%)
Ustekinumab	70 (10%)
Tofacitinib	131 (18%)
Filgotinib	130 (18%)
Upadacitinib	32 (4%)
Ozanimod	4 (< 1%)

Therapy class exposure	All patients *n* = 720
Anti‐TNF	433 (60%)
JAK inhibitor	265 (37%)

Abbreviations: Anti‐TNF, anti‐tumour necrosis factor; JAK, janus kinase.

^a^ Previous colonic surgery excluding subtotal colectomy and panproctocolectomy.

^b^ Mesalazine exposure available for 701 patients.

^c^ Thiopurine exposure available for 700 patients.

**TABLE 2:** Colectomy cohort demographics and phenotype at time of surgery.

Demographic	All patients *n* = 563
Male sex, *n* (%)	324 (58%)
Age at diagnosis, years, median (IQR)	33 (24, 47)
Age at colectomy, years, median (IQR)	42 (29, 56)
Time from diagnosis to colectomy, years, median (IQR)	2.6 years (0.4, 9.4)
Time from first advanced therapy to colectomy, median (IQR)	8.9 months (IQR 1–31)
Paediatric population, *n* (%)	17 (3%)
Elderly population, *n* (%)	65 (12%)
Montreal Classification^a^, *n* (%)	
E1	10 (2%)
E2	167 (32%)
E3	343 (66%)
Colectomy within 90 days of diagnosis, *n* (%)	114 (22%)^b^
Emergency colectomy, *n* (%)	359 (68%)^c^
Underwent a subtotal colectomy, *n* (%)	420 (81%)^d^
Underwent a pan proctocolectomy, *n* (%)	99 (19%)^d^

Advanced therapy class exposure, *n* (%)	All patients *n* = 563
Any class	138 (25%)
Anti‐TNF	129 (23%)
Anti‐integrin	51 (9%)
IL‐12/23 p40 subunit inhibitor	17 (3%)
JAK inhibitor	24 (4%)
S1P receptor modulator	0

Number of advanced therapies prior to colectomy, *n* (%)	All patients *n* = 563
One	85 (15%)
Two	26 (5%)
Three	13 (2%)
Four	7 (1%)
Five	7 (1%)

Abbreviations: Anti‐TNF, anti‐tumour necrosis factor; IL, interleukin; JAK, janus kinase; S1P, Sphingosine‐1 phosphate.

^a^ 38 patients from 2004 complete histology reports unavailable.

^b^ 30 patients unclear time of colectomy from diagnosis.

^c^ 36 patients unclear urgency of procedure.

^d^ 44 patients primary operation was unclear.

### 3.2 Advanced Therapy Prescribing

**FIGURE 1:**
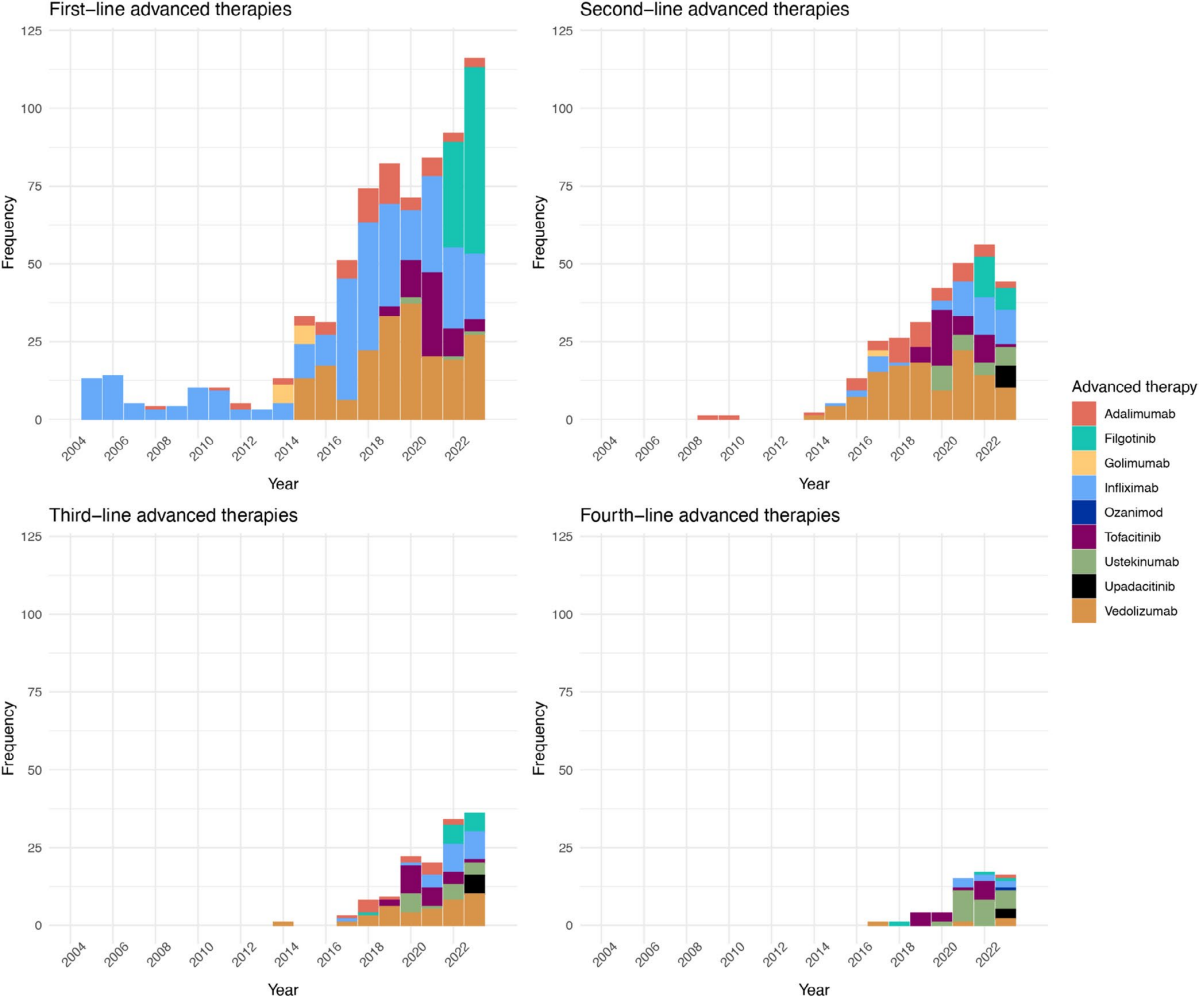
Total number of new advanced therapy prescriptions in the Lothian UC population stratified by year. Number of patients per group; first‐line therapies = 720, second‐line therapies = 297 (41%), third‐line therapies = 130 (18%) and fourth‐line therapies = 59 (8%). A total of 25 patients (3%) had more than four lines of advanced therapies.

**FIGURE 2:**
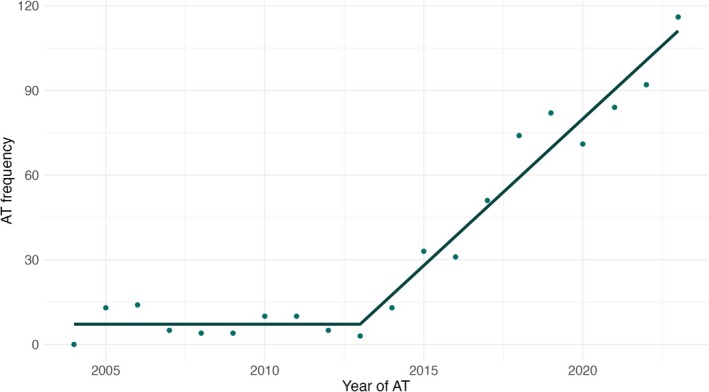
Total number of first advanced therapies prescribed per year in the Lothian UC population stratified by time from 2004 to 2023. A total of 716 first advanced therapies were prescribed across the study period. Piecewise linear regression produced a breakpoint at the beginning of 2013 for first advanced therapy prescriptions and an average annual percent change of 5.47 (95% CI: 4.83, 6.11).

**FIGURE 3:**
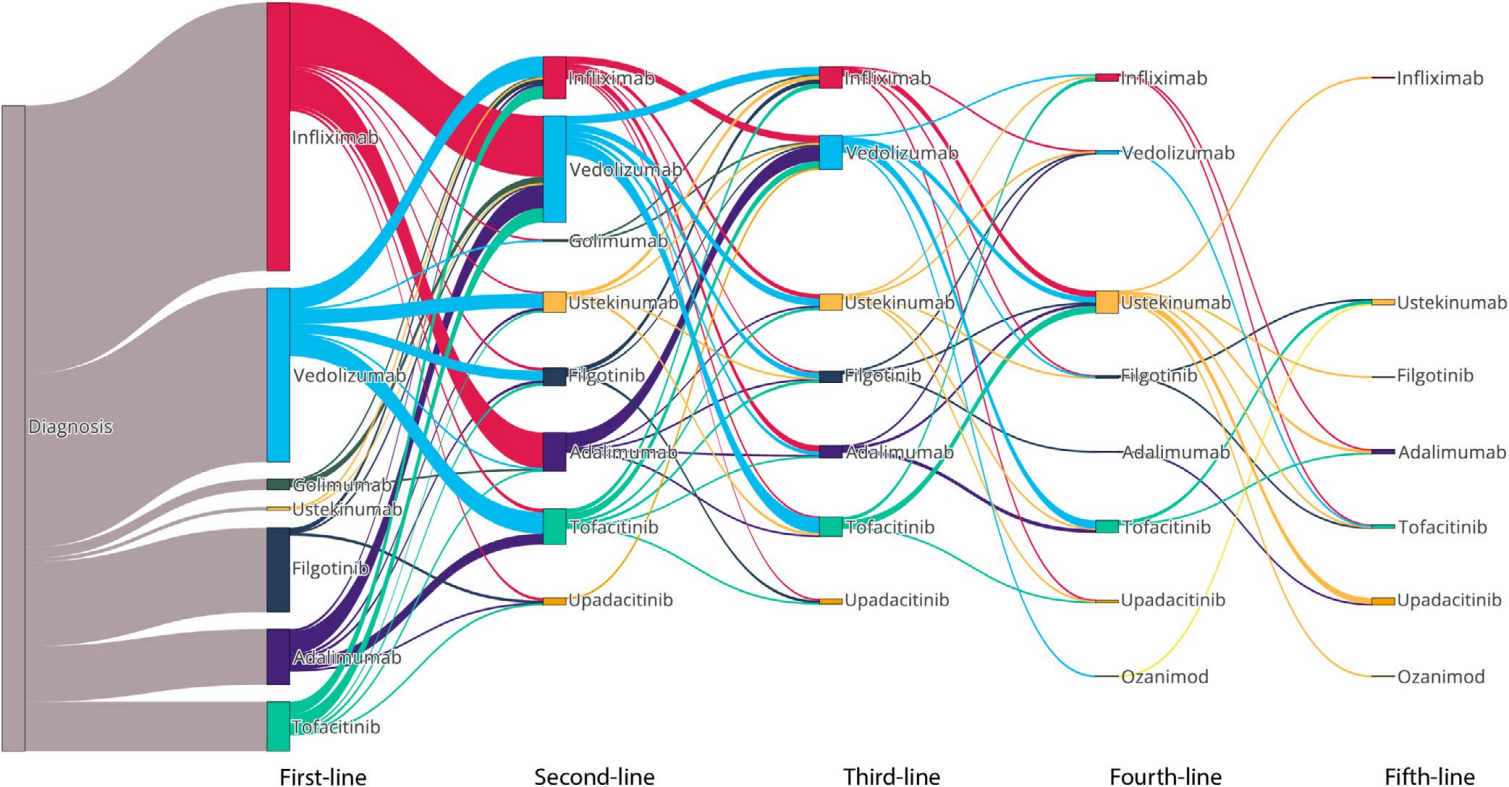
Sankey diagram demonstrating treatment progression from first‐line therapy through to subsequent lines of therapy in the Lothian UC population.

**FIGURE 4:**
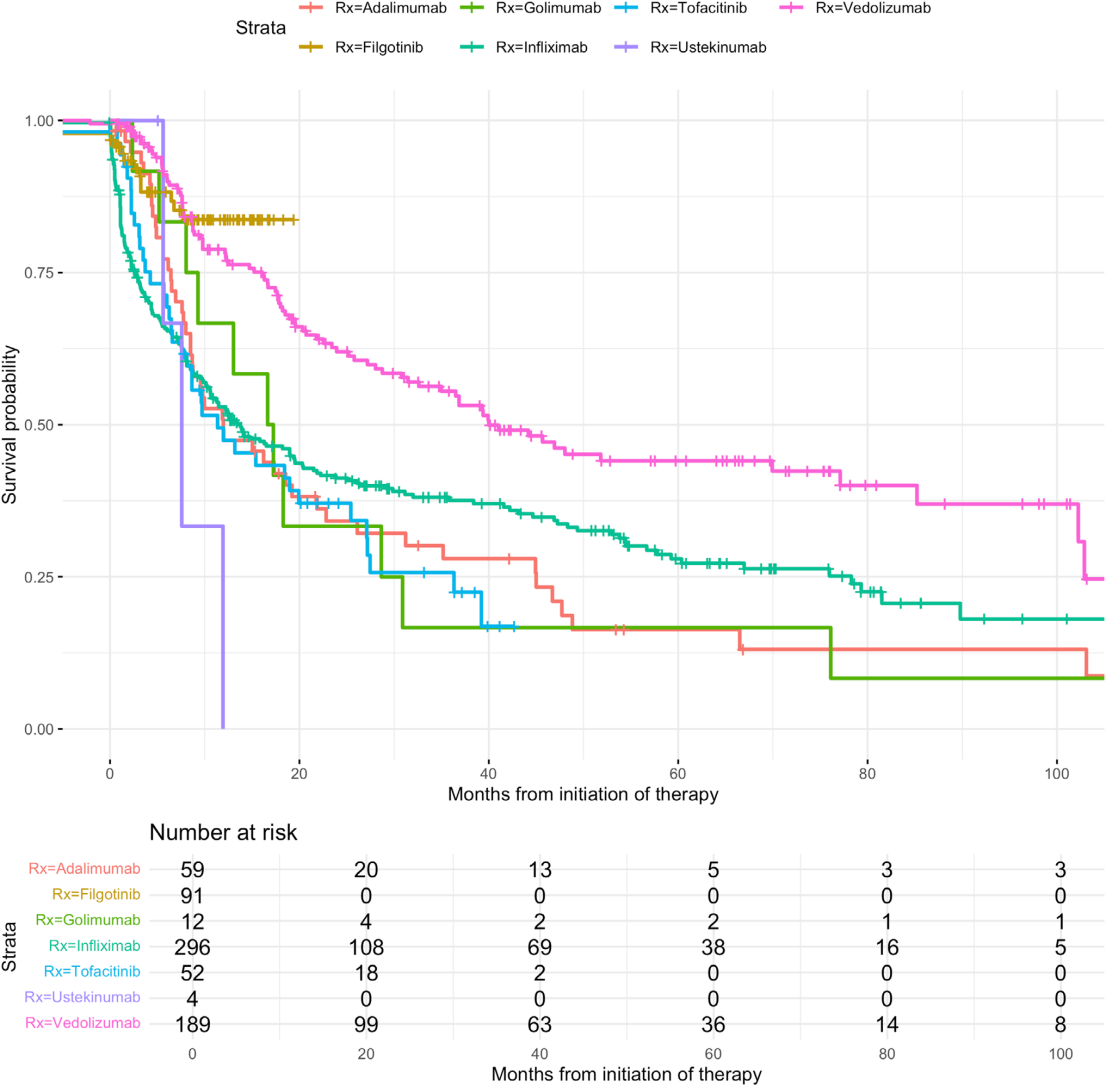
Kaplan–Meier curve demonstrating first‐line treatment persistence amongst patients who commenced an advanced therapy for UC in Lothian.

First‐line advanced therapy prescribing per 100 UC patients increased from 0.00 in 2004 to 2.82 in 2023 (Figure 1). Piecewise linear regression produced a joinpoint at the beginning of 2013 for first advanced therapy prescriptions, and an average annual percent change of 5.47 (95% CI: 4.83, 6.11) was observed (Figure 2). In the first segment (2004–2013), an annual percent change of 0 (95% CI: −1.7472, 1.7462) was observed. In the second segment (2013–2023), an annual percent change of 10.391 (95% CI: 8.644, 12.137) was observed. At the conclusion of 2023, 41% (297/720), 18% (130/720), 8% (59/720) and 3% (24/720) of the 720 patients who were advanced therapy exposed had received second, third, fourth and fifth‐line advanced therapy, respectively. Infliximab was the most prescribed first‐line therapy (42%, 299/720) and vedolizumab was the most commonly prescribed second‐line (41%, 119/288) and third‐line therapy (29%, 38/130) (Figure 3). From 2019 onwards, similar proportions of infliximab, vedolizumab and JAK inhibitors were used as first‐line treatment for UC (Figure S4). In biologic naïve elderly patients (age ≥ 65 years old), vedolizumab was the most commonly prescribed first‐line therapy (74%, 49/66), with ustekinumab (33%, 10/30) and vedolizumab (27%, 9/30) the most common second‐line therapies. Vedolizumab and filgotinib had the highest one‐year persistence rates (75% vs. 68%); however, filgotinib‐treated patients had a shorter duration of follow‐up (< 2 years) (Figure 4).

We observed that 60% (217/359) of initial infliximab treatments were used as ASUC therapy (Figure S5). ASUC infliximab therapy was successful in preventing death or colectomy during that admission in 89% (194/217) of patients, with death occurring in < 5 patients. Colectomy‐free survival decreased to 80% (174/217) at 12 months for those who required infliximab for ASUC. Successful ASUC therapy did not continue in the ambulatory care setting in 19% (36/194) of patients, predominantly due to restricted access to infliximab maintenance therapy prior to 2015.

### 3.3 Colectomy Results

**FIGURE 5:**
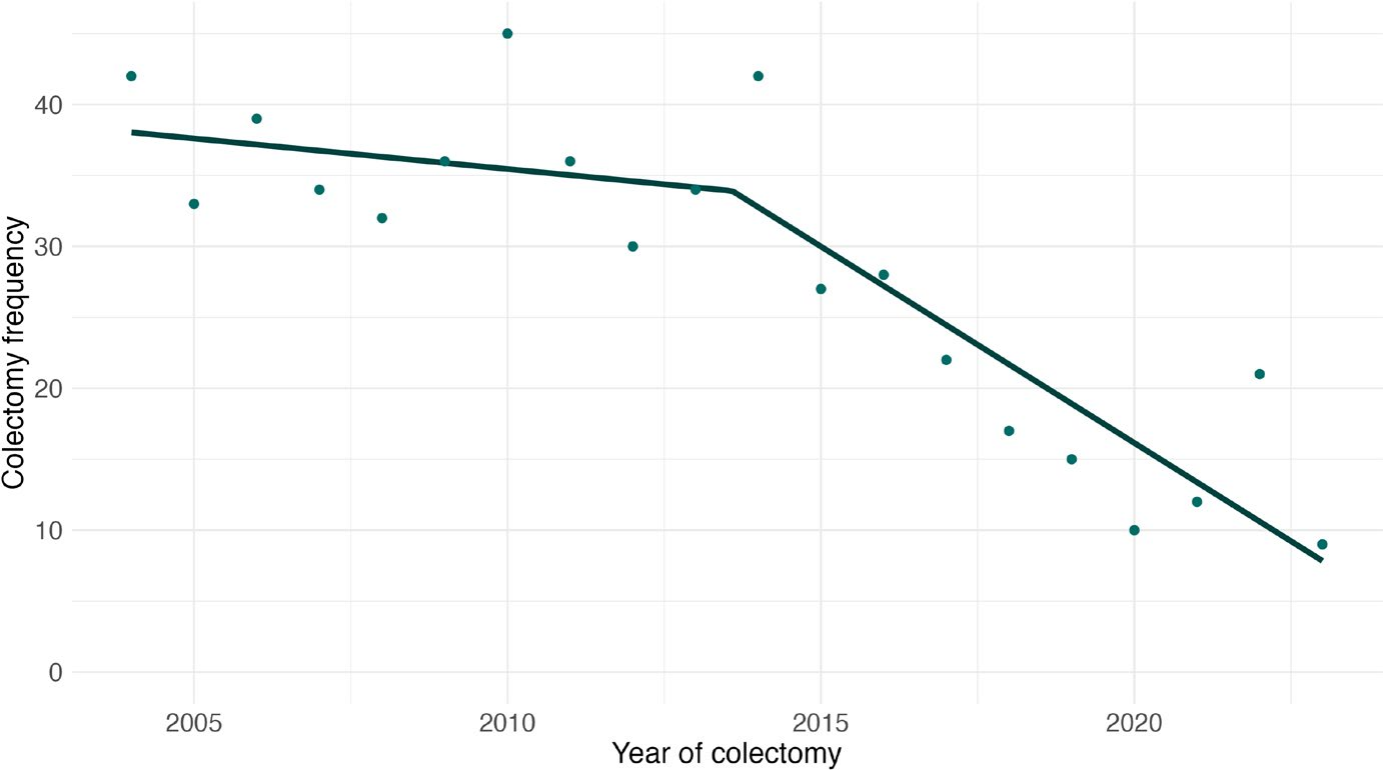
Total number of colectomies per year in the Lothian UC population stratified by time from 2004 to 2023. A total of 563 colectomies were performed across the study period. Piecewise linear regression produced a breakpoint mid 2013 for colectomies and an average annual percent change of −1.59 (95% CI: −2.05, −1.13) was observed.

**FIGURE 6:**
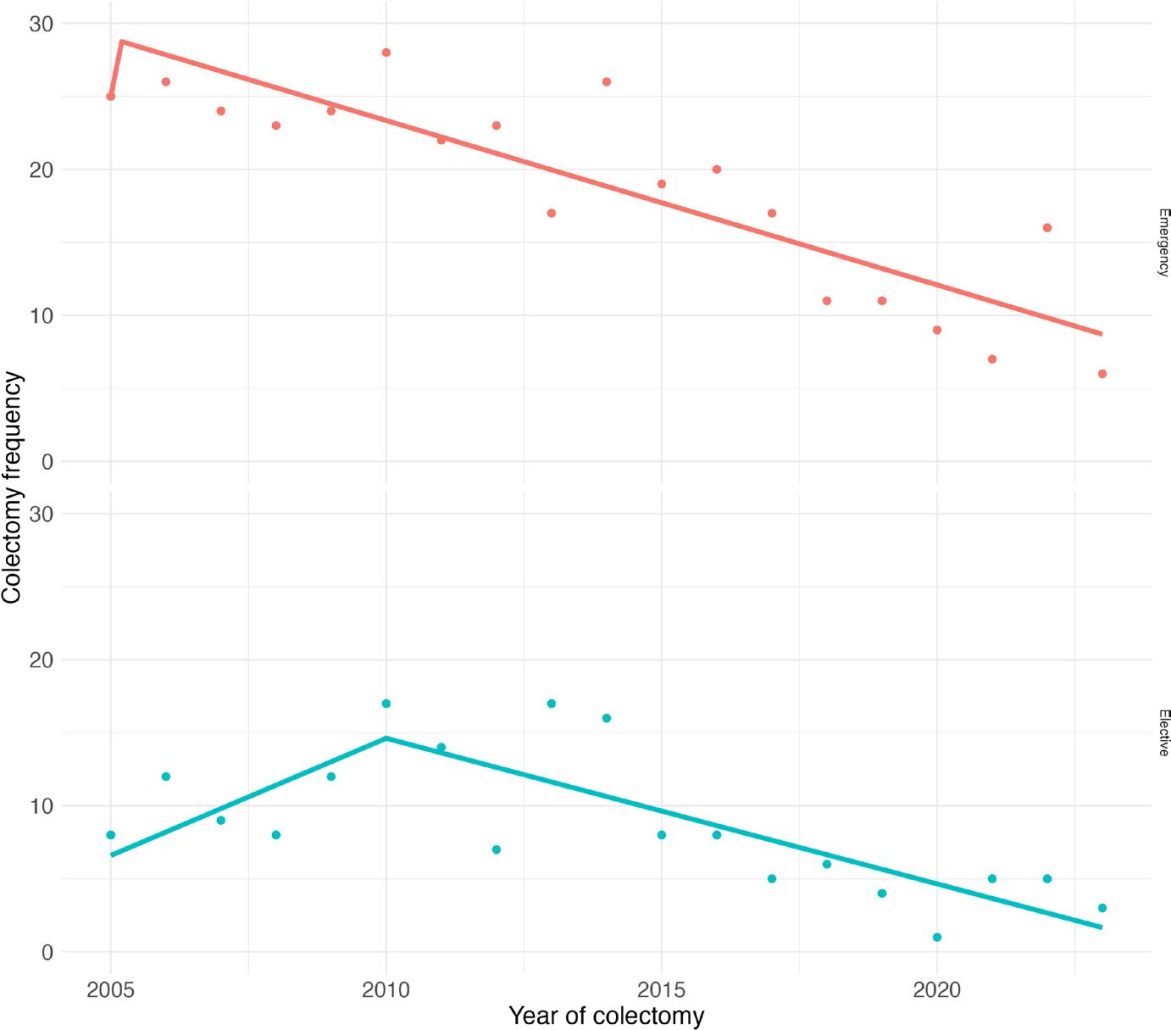
Total numbers of elective and emergency colectomies in the Lothian UC population stratified per year. Emergency colectomy = 359 (68%, 359/527). Elective colectomy = 168 (32%, 168/527). Urgency of operation data unavailable for 36 patients. Piecewise linear regression produced a breakpoint in 2005 for emergency colectomies (APC of 0.14, 95% CI of [−0.26, 0.55]) and 2010 for elective colectomies (APC of −0.18 95% CI of [−0.46, 0.11]).

Colectomy rates per 100 UC patients decreased from 2.48 in 2004 to 0.22 in 2023 (Figure 5). Emergency colectomy rates per 100 UC patients decreased from 1.71 in 2005 to 0.22 in 2023 and elective colectomy rates per 100 UC patients decreased from 1.47 in 2005 to 0.05 in 2023 (Figure 6). Piecewise linear regression produced a breakpoint mid 2013 for colectomies and an average annual percent change of −1.59 (95% CI: −2.05, −1.13) was observed. In the first segment (2004–2013), an annual percent change of −0.43 (95% CI: −1.69, 0.83) was observed. In the second segment (2013–2023), an annual percent change of −2.77 (95% CI: −4.03, −1.51) was observed. The model for non‐elective surgery has an average annual percent change of 0.14 (95% CI of [−0.26, 0.55]). The annual percent change for the first segment (2004–2005.) is 19.00 (95% CI of [8.26, 29.74]). The annual percent change for the second segment (2005–2023) is −1.13 (95% CI of [−1.47, −0.78]). The model for elective surgery has an average annual percent change of −0.18 (95% CI of [−0.46, 0.11]). The annual percent change for the first segment (2004–2010) is 1.61 (95% CI of [0.32, 2.89]). The annual percent change for the second segment (2010–2023) is −1.00 (95% CI of [−1.50, −0.49]).

Elderly patients represented 12% (65/564) of the colectomy cohort and paediatric patients (age ≤ 16 years old) represented 3% (17/564) of colectomies, of which there were no colectomies for paediatric patients from 2016 to 2023. We observed that 65% (344/526, missing extent = 38) of colectomies occurred in patients with extensive disease (Montreal E3), 32% (167/526) with left‐sided disease (E2), and 2% (10/526) with proctitis (E1), with 68% (360/532) of colectomies performed for ASUC, of which 31% (112/360) occurred within 90 days of diagnosis. A drop in emergency colectomies occurred for both early diagnoses (from 0.16 per 100 UC patients in 2005 to 0.07 per 100 in 2023), and established disease (from 1.13 per 100 UC patients in 2004 to 0.07 per 100 in 2023) (Figure S6). A total of 4% (24/563) of colectomies were for cancer (*n* = 15) or dysplasia/suspected cancer (*n* = 9). Colectomies for ‘chronic active UC refractory to medical treatment’ have almost completely disappeared with only 5 colectomies performed for this indication in the last 2 years of follow‐up (Figure S7).

We observed that 81% of the cohort underwent a subtotal colectomy and 19% underwent a pan proctocolectomy for their initial surgery. Rates of IPAA formation were 67.6% (303/448) in the 2005–2018 cohort and 60% (25/42) in the updated 2019–2023 cohort (20 patients had not yet made a decision regarding IPAA vs. permanent ileostomy in the updated cohort).

We observed that 75% (425/563) of the colectomy cohort did not receive an advanced therapy prior to colectomy. The proportion of patients who did not receive an advanced therapy prior to a colectomy was 100% (42/42) in 2004 and < 5/30 from 2022 to2023. We observed that 10% (54/563) of patients had multiple advanced therapies prior to colectomy (Table 2). From 2022 to 2023, 83% (25/30) had received an anti‐TNF therapy prior to colectomy, and 50% (15/30) had received both an anti‐TNF and a JAKi prior to colectomy. Colectomy within 7 days of first advanced therapy occurred in 4% (24/563) of the total colectomy cohort and 22% (24/107) amongst those who received an advanced therapy prior to colectomy.

### 3.4 Colectomy Complications

Total complications within the first 30 days of the colectomy occurred in 39% (150/382) of patients, major complications occurred in 12% (44/382) of patients, and infective complications within 30 days of colectomy occurred in 18% (69/382) of patients (Table S1). Both infective and non‐infective complication rates were stable across the study period (Figure S8). Rates of infective complications were lower for those receiving an advanced therapy (14%, 19/135) compared to those who were not (20%, 50/247). Death within 30 days occurred in 1.6% (6/382) of colectomies, predominantly in older comorbid patients.

## 4 Discussion

In a population‐based cohort with a high prevalence of UC spanning twenty years, we observed that advanced therapy prescribing rates increased for both first‐line therapy and subsequent lines of therapy. We observed an increase in advanced therapy prescribing from our previous results in 2018 and a substantial decrease in colectomy rates. Piecewise linear regression found a joinpoint in 2013 for both increased advanced therapy prescribing and decreased colectomy rates. Whilst association cannot ascribe causality, we found more frequent advanced therapy use, along with multiple different therapies, has co‐occurred with lower rates of colectomy.

Our results demonstrate the recent expansion of therapeutic options for UC. This has resulted in patients receiving more lines of therapy. In the last two years, we observed a switch to filgotinib as the most used first‐line therapy which is significant given it is an oral small molecule. This has ramifications for healthcare delivery, decreasing the need for infusion beds or subcutaneous injection delivery training, therefore saving money and resources. This is a trend which looks likely to continue with increased use of S1PR modulators for early lines of therapy [16].

Data in the literature describing advanced prescribing trends in UC are predominantly limited to biologics, in particular anti‐TNFs and vedolizumab, much like our previously published cohort [17, 18, 19, 20, 21, 22]. Data compiled from a large US commercial database of healthcare records observed a large increase in biologic use from 0.5% in 2000 to 12.8% in 2019 [18]. Population health studies in South Korea and Israel have demonstrated a substantial uptake in biologic therapies (0.7% in 2010 to 8.2% in 2018 in South Korea [19], and 0.1% in 2005 to 9.6% 2019 in Israel) [21]. The earlier and more frequent use of biologic therapies has been associated with decreased corticosteroid use with patients on biologics [17], and lower corticosteroid use in general amongst the UC population [19]. The regular upfront advanced therapy prescriptions of both vedolizumab and infliximab in Lothian from 2015 to 2021 stand in contrast to the Danish experience from 2015 to 2020, where 89% of first therapy was infliximab [23], and the UK IBD bioresource cohort where infliximab was used 70% first‐line [24]. This may have significance given vedolizumab had the best persistence rates in our Lothian cohort [25], as well as Danish [23], UK [24], and Australian cohorts [26].

We observed a reduction in the number of colectomies performed each year as demonstrated by the colectomy rate change from 2.48 per 100 UC patients in 2004 to 0.22 in 2023. We observed a similar reduction occurring across all clinical scenarios, including shortly after diagnosis, hospitalisation, and medically refractory elective patients. This represents an important contribution to the literature given colectomy rates in UC data from across the world are conflicting with different methods data collection such as population and hospital‐based data [5, 6, 7, 8].

Population studies from Israel [21], and Hungary (1977–2020) [7] observed no decrease in colectomy rates in the biologic era despite increased biologic use. A South Korean population study observed a drop in colectomy rates from 0.4% in 2010 to 0.2% in 2014, which then stabilised through to the end of 2018 despite the increasing uptake of biologics [19]. In somewhat similar fashion, data from a major Italian tertiary referral centre from 1960 to 2013 observed reduction in colectomy rates, but this did not correlate with the introduction of anti‐TNF therapy [27]. Data from the IBSEN III inception cohort suggests early use of advanced therapy with a ‘treat‐to‐target’ approach reduces 1‐year colectomy rates [28]. The IBSEN III inception cohort was formed from patients diagnosed with UC from 2017 to 2019, and were prospectively followed up for 1‐year. A relatively high proportion of the cohort (14.5%) received biologic therapy within 1 year of diagnosis. Colectomy rates were 0.9% at 1‐year, which is lower than their colectomy rates of 3.5% in their pre‐biologic era IBSEN cohort [29]. A meta‐analysis published in 2021 on colectomy rates from population studies found a decrease in colectomy rates in the biologic era (2004 onwards), with a relative risk of colectomy 0.71 at 5 years post diagnosis [30]. This analysis did not correlate outcomes with advanced therapy prescribing trends and does not factor for the recent additions to the UC treatment armamentarium.

There are also conflicting findings regarding the effect of advanced therapies on emergency and elective colectomy rates. Nationwide population data from England (2003–2016) [5], and Japan (2007–2017) [20] observed a reduction in total and elective colectomy rates but not emergency rates. However, multicentric inpatient sample database data from the US (2007–2016) observed a decrease in total colectomies and emergency colectomies; however, there was an increase in the proportion and total number of elective colectomies [6]. Our data demonstrate a drop in both emergency and elective colectomies, with the proportion of elective and emergency colectomies similar throughout the study period. Our data suggest a change in the natural history of UC throughout the biologic era and may be explained by better treatment. In Lothian, the increased uptake of advanced therapies has corresponded to lower surgical rates in the Crohn's disease population [31], as well as a 36% decrease in hospital admission rates from 2010 to 2019, providing additional evidence that increasing advanced therapy prescribing is associated with better outcomes in the IBD population [32]. One of the reasons we may have observed lower colectomy rates in all scenarios, as compared to some other regions, is our longer length of follow‐up during the biologic era enabling us to more confidently measure the effect of new treatments and therapeutic sequencing. Many of the aforementioned studies concluded prior to 2019 and could not adequately assess the effect of the additional new classes of therapy that have subsequently emerged. Our cohort had a substantial proportion of patients who commenced either vedolizumab or a JAK inhibitor as first‐line therapy. This is unique amongst the previously prescribed sequencing cohorts and may have affected colectomy rates.

One concern with the increasing use of advanced therapies is the potential negative immunosuppressive effects on surgical complications rates. We did not observe higher total or infective complication rates amongst those receiving an advanced therapy compared. These findings are consistent with those of the PUCCINI cohort, where pre‐operative anti‐TNF exposure in IBD patients undergoing abdominal surgery was not associated with an increased post‐operative infection rate [33], and a meta‐analysis published in 2020 examining pre‐operative biologic therapy effect on post‐operative complication rates in UC, which did not find an increased risk of complications with biologic therapy [34].

The key strengths of our study are that it is a population‐based study with manually confirmed individual patient level data for both paediatric and adult patients, and analyses performed using rigorously validated prevalence rates of UC. Our data is timely given that data regarding small molecules and ustekinumab are not included in most studies on this topic in the literature. Limitations include the retrospective nature of the study, and the study was done in one region. Descriptive data regarding mesalazine, thiopurine use, and corticosteroids were unavailable for analysis given the multiple avenues for prescribing (primary, emergency and tertiary care) and were not considered an aim for the study, given that corticosteroids have been shown to be ineffective in maintaining remission [12]. Lothian is a research and resource‐rich setting with ready access to advanced therapies, and these data may not be generalisable to less resource‐rich settings, regions with different therapeutic sequencing, or regions with reduced access to advanced therapies. Whilst there is a clear correlation between the uptake of advanced therapies and decreased colectomy rates over time, correlation cannot ascribe causation. Regarding colectomy complications, corticosteroid prescribing data, comorbidities, length of time to surgery, both elective waiting times, and time from admission for emergency colectomies were not available.

In conclusion, there have been significant changes in prescribing trends over the last 20 years in UC, with a particular uptake in the proportion of patients with UC treated with an advanced therapy, and the number of lines of advanced therapies they are receiving. Over this same time period, colectomy rates for UC dropped, reflecting a change in UC disease course.

## Author Contributions

**Alexander T. Elford:** conceptualization, methodology, formal analysis, data curation, writing – original draft, writing – review and editing, project administration, investigation, visualization. **Nathan Constantine‐Cooke:** writing – review and editing, formal analysis, software. **Phil W. Jenkinson:** writing – review and editing, methodology, conceptualization. **Beatriz Gros:** data curation, writing – review and editing. **Nikolas Plevris:** writing – review and editing, methodology. **Mathew Lyons:** methodology, writing – review and editing. **Solomon Ong:** data curation, writing – review and editing. **Neil Greenlees:** data curation, writing – review and editing. **Victor Velasco‐Pardo:** formal analysis, writing – review and editing. **Nicholas T. Ventham:** writing – review and editing, methodology. **Paul Henderson:** writing – review and editing, methodology. **David C. Wilson:** methodology, writing – review and editing. **Shahida Din:** methodology, writing – review and editing. **Colin L. Noble:** methodology, writing – review and editing. **Gareth‐Rhys Jones:** methodology, writing – review and editing, supervision. **Ian Arnott:** methodology, writing – review and editing, supervision. **Charlie W. Lees:** conceptualization, methodology, formal analysis, writing – review and editing, supervision. **Claire O'Hare:** data curation, investigation.

## Disclosure

Alexander Elford has received travel expenses support from Dr. Falk Pharma, Ferring Pharmaceuticals and Galapagos. Nathan Constantine‐Cooke, Neil Greenlees, Victor Velasco‐Pardo, Solomon Ong, Nicholas Ventham, Paul Henderson and David Wilson have no disclosures. Beatriz Gros has served as a consultant to Abbvie, Pfizer, Roche and Galapagos and has served as a speaker for Abbvie, Janssen, Takeda, Pfizer, Roche and Galapagos. Nikolas Plevris has served as a speaker for Abbvie, Janssen, Pfizer and Fresenius Kabi and served as a consultant for Abbvie and Janssen. Shahida Din reports consulting fees from Abbvie, personal speaker fees from Janssen, Takeda, Ferring and meeting and travel grants from Janssen, Takeda, Lily, Abbvie and Dr. Falk. Colin Noble has done advisory work for Galapagos. Gareth‐Rhys Jones has received speaking fees for Janssen, Ferring, Fresenius, Takeda and Abbvie. Ian Arnott's disclosures include Galapagos/Alpha sigma, Vifor, Abbvie, Lily, BMS and Pfizer. Charlie Lees has acted as a consultant to Abbvie, Janssen, Takeda, Pfizer, Galapagos, Eli Lilly, Bristol Myers Squibb, B.I., Sandoz, Novartis, GSK, Gilead, ViforPharma, Dr. Falk and Iterative Health; he has received speaking fees and travel support from Pfizer, Janssen, Abbvie, Eli Lilly, Galapagos, MSD, Takeda, Shire, Ferring, Hospira and Dr. Falk.

## Supporting information


Data S1.

## Data Availability

The data that support the findings of this study are available on request from the corresponding author. The data are not publicly available due to privacy or ethical restrictions.
