# Supplementary material for: Twenty‐Year Trends in Colectomy Rates and Advanced Therapy Prescribing in Lothian, Scotland
Source: Aliment Pharmacol Ther. 2025 Jun 16;62(7):699–709. doi: 10.1111/apt.70240 (PMC12422722; doi:10.1111/apt.70240)
Supplement: Supplementary file 1 — Data S1. [file APT-62-699-s001.docx]

**Supplemental Figure 1-** Flow chart illustrating the inclusion of patients in the advanced therapy cohort from the Lothian UC population. Alternate IBD subtypes included IBD-unclassified and Crohn’s disease. Transient residents were those who received an advanced therapy in Lothian for less than 12 months.

**
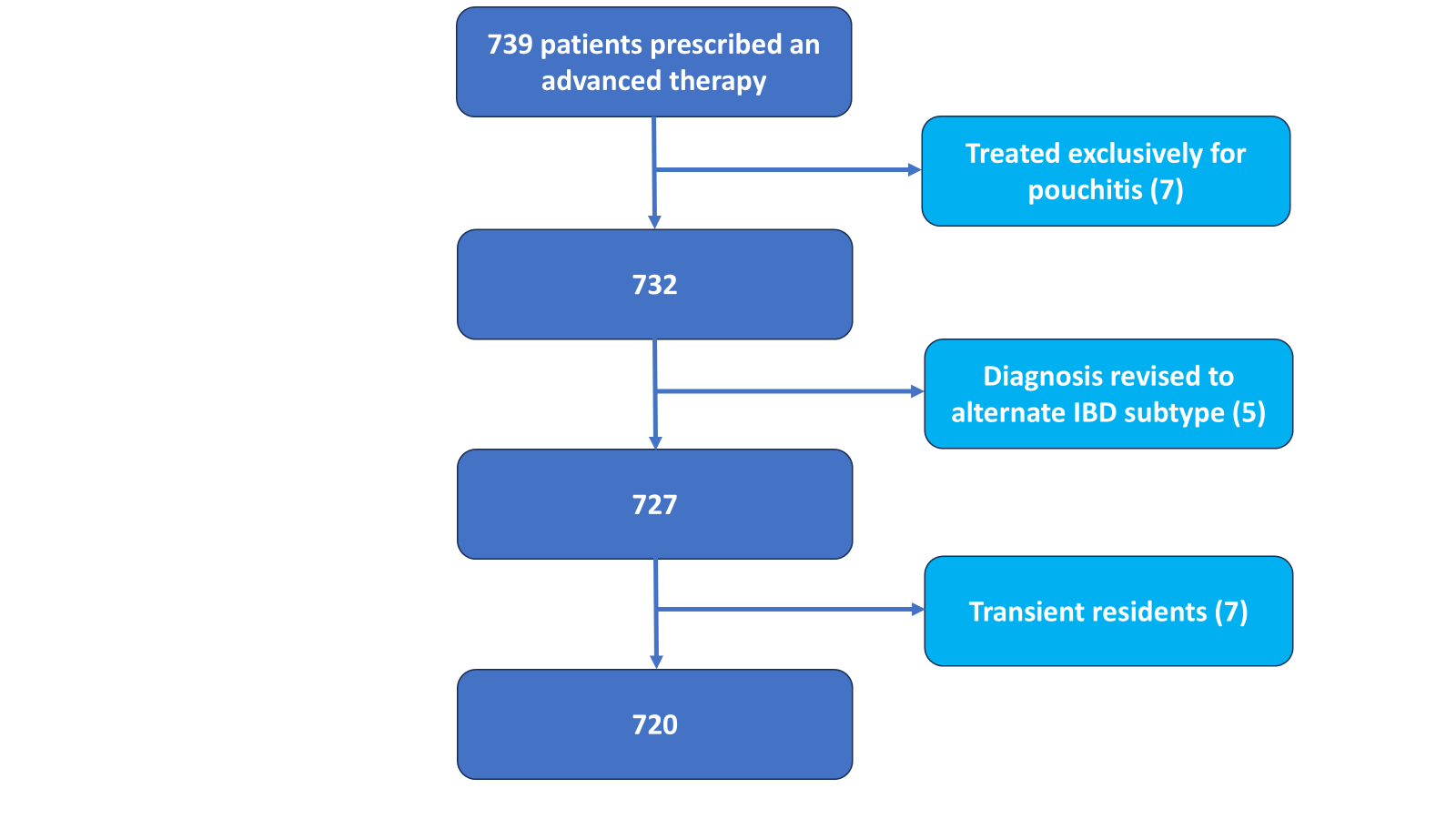
**

IBD, inflammatory bowel disease.

**Supplemental Figure 2-**  Distribution of time to first advanced therapy by time of advanced therapy commencement from 2015, when biologics became routinely available for maintenance therapy. Outliers have been removed to avoid presenting individual-level data.


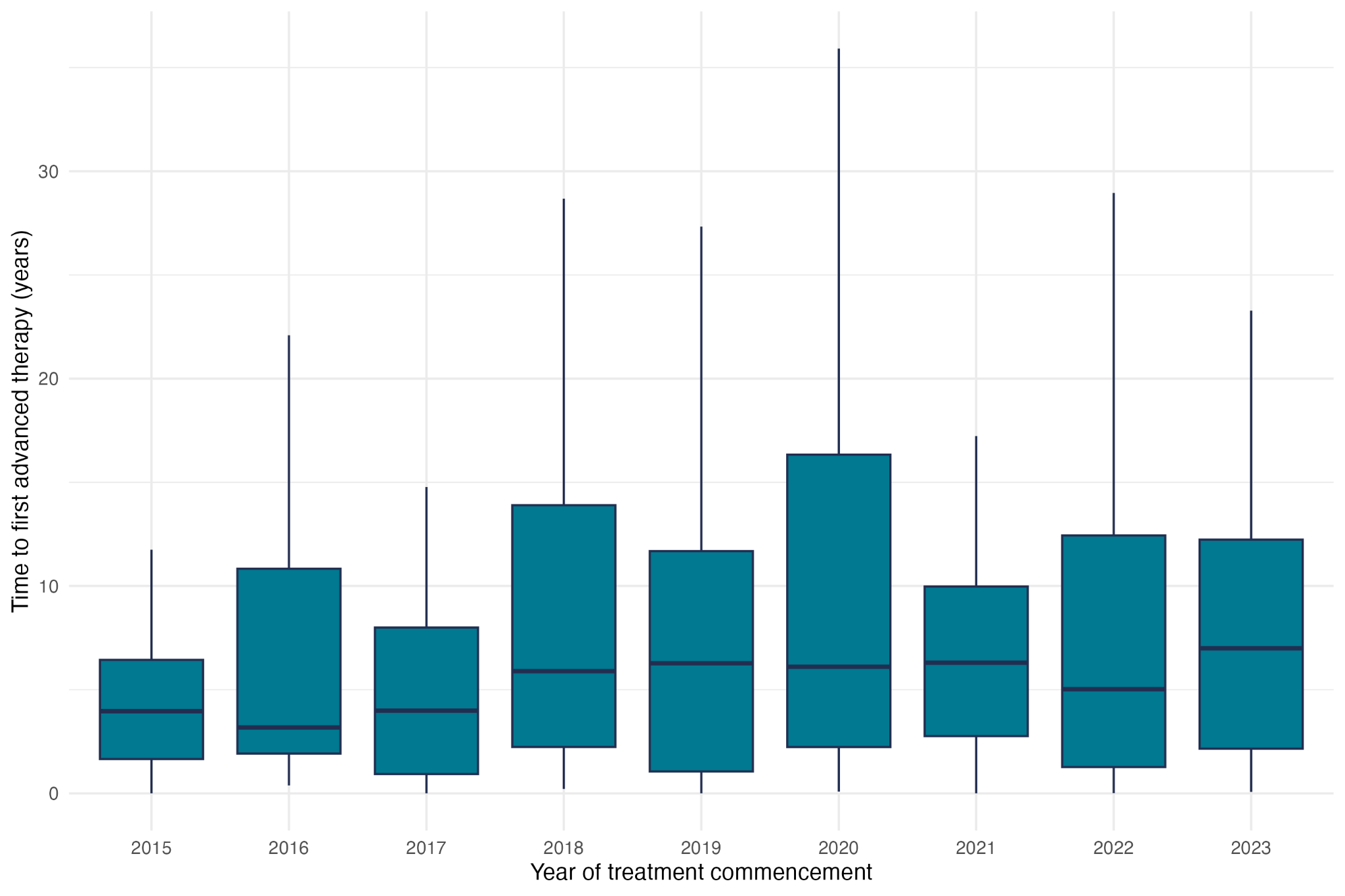


**Supplemental Figure 3-**  Distribution of time from diagnosis to colectomy by year in the Lothian UC cohort. Outliers have been removed to avoid presenting individual-level data.

**
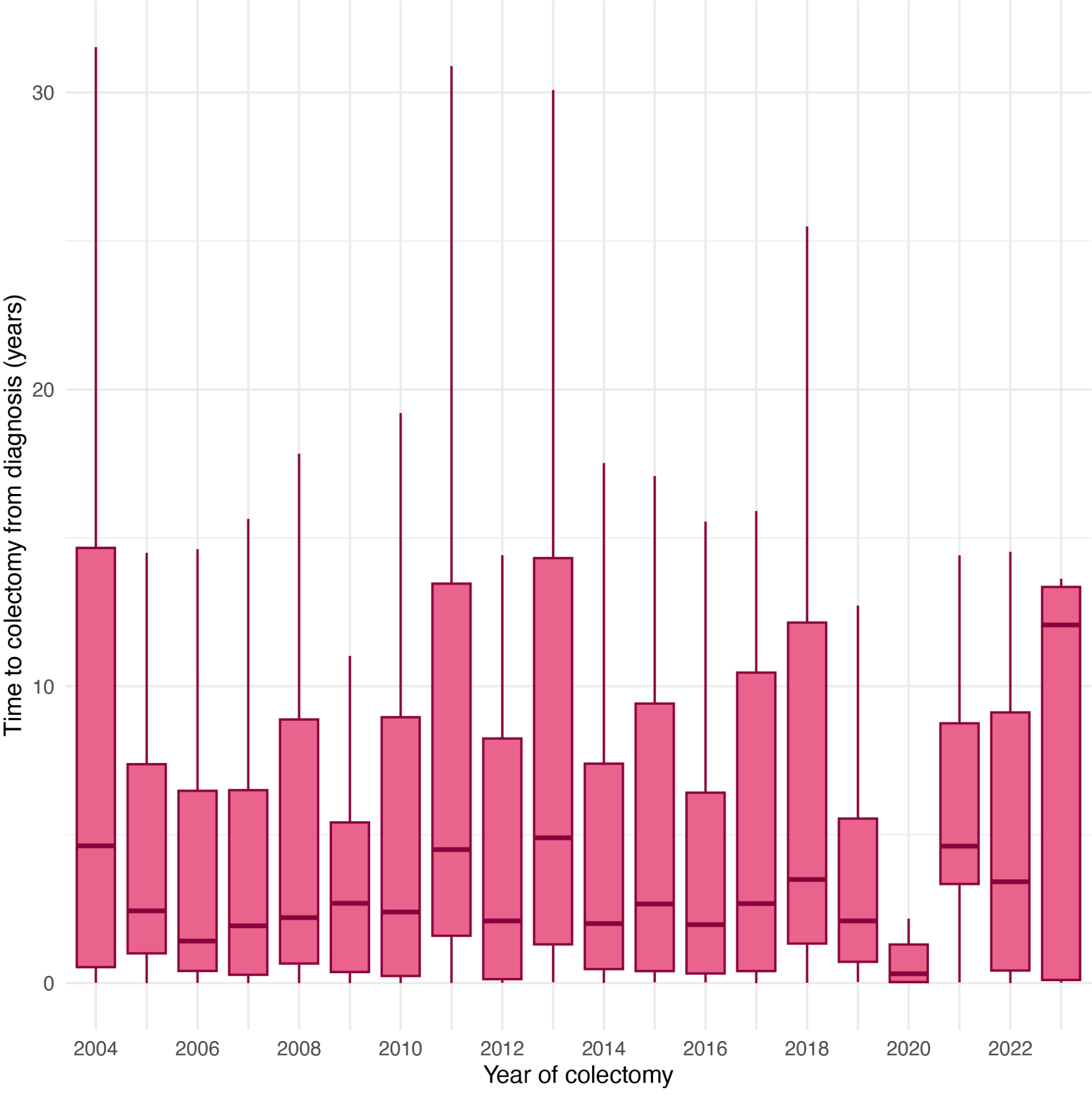
**

**Supplemental Figure 4-** Sankey diagram demonstrating treatment progression in the Lothian UC population for (A) subjects who started their first advanced therapy before 2019 and (B) subjects who started their first advanced therapy in 2019 or later. It should be noted the median follow-up from start of first advanced therapy is 5.9 years in (A) and 1.8 years in (B).

**
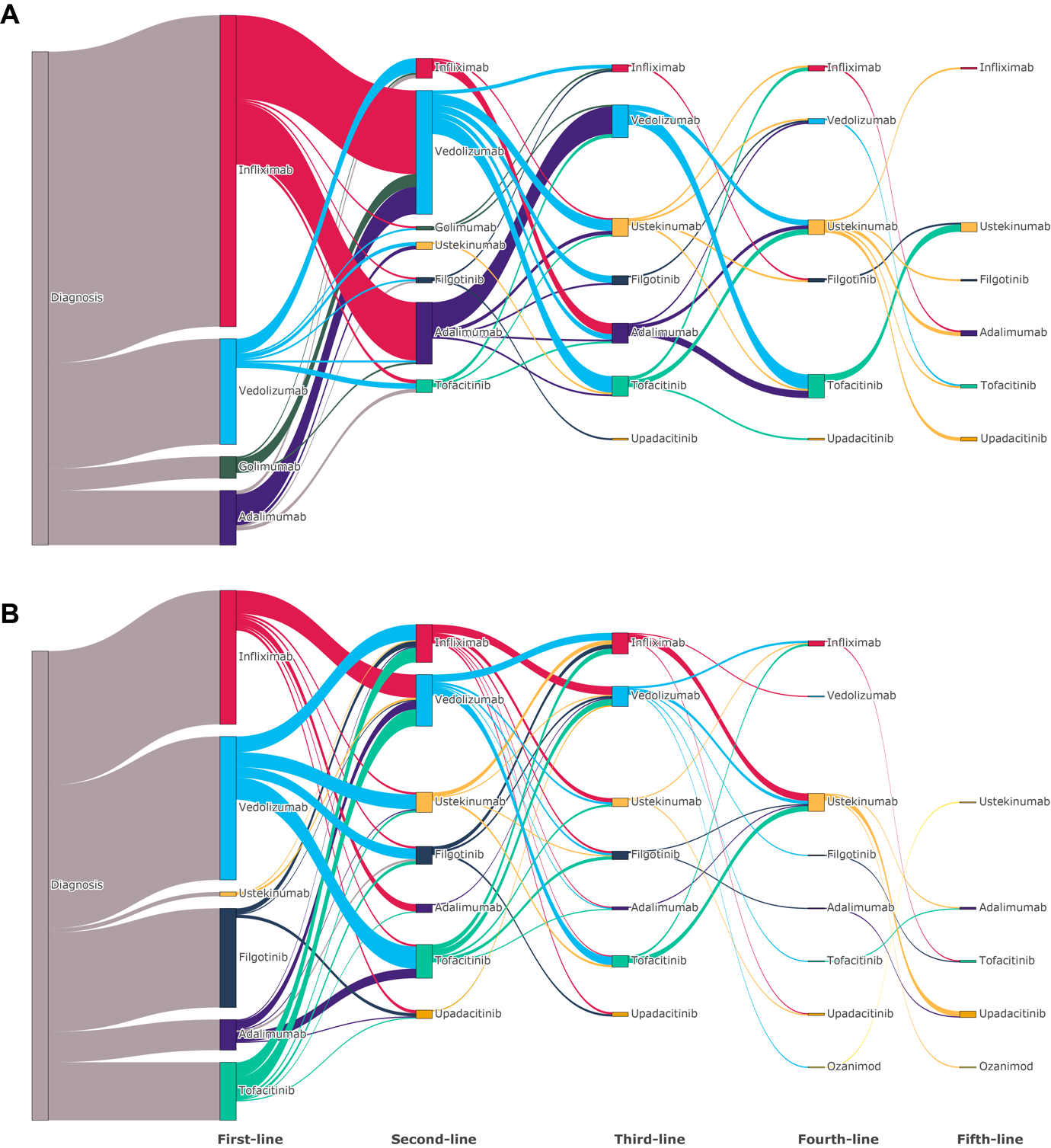
**

**Supplemental Figure 5-** Breakdown of infliximab prescribing for the Lothian UC population stratified by initial indication and success. Rescue acute severe UC (ASUC) therapy was defined as therapy initially commenced as inpatient salvage therapy. Outpatient treatment was defined as infliximab use commenced in the ambulatory care setting. The 23 patients with unsuccessful rescue ASUC therapy required colectomy or died.*Initial indication data unavailable for twelve patients.

**
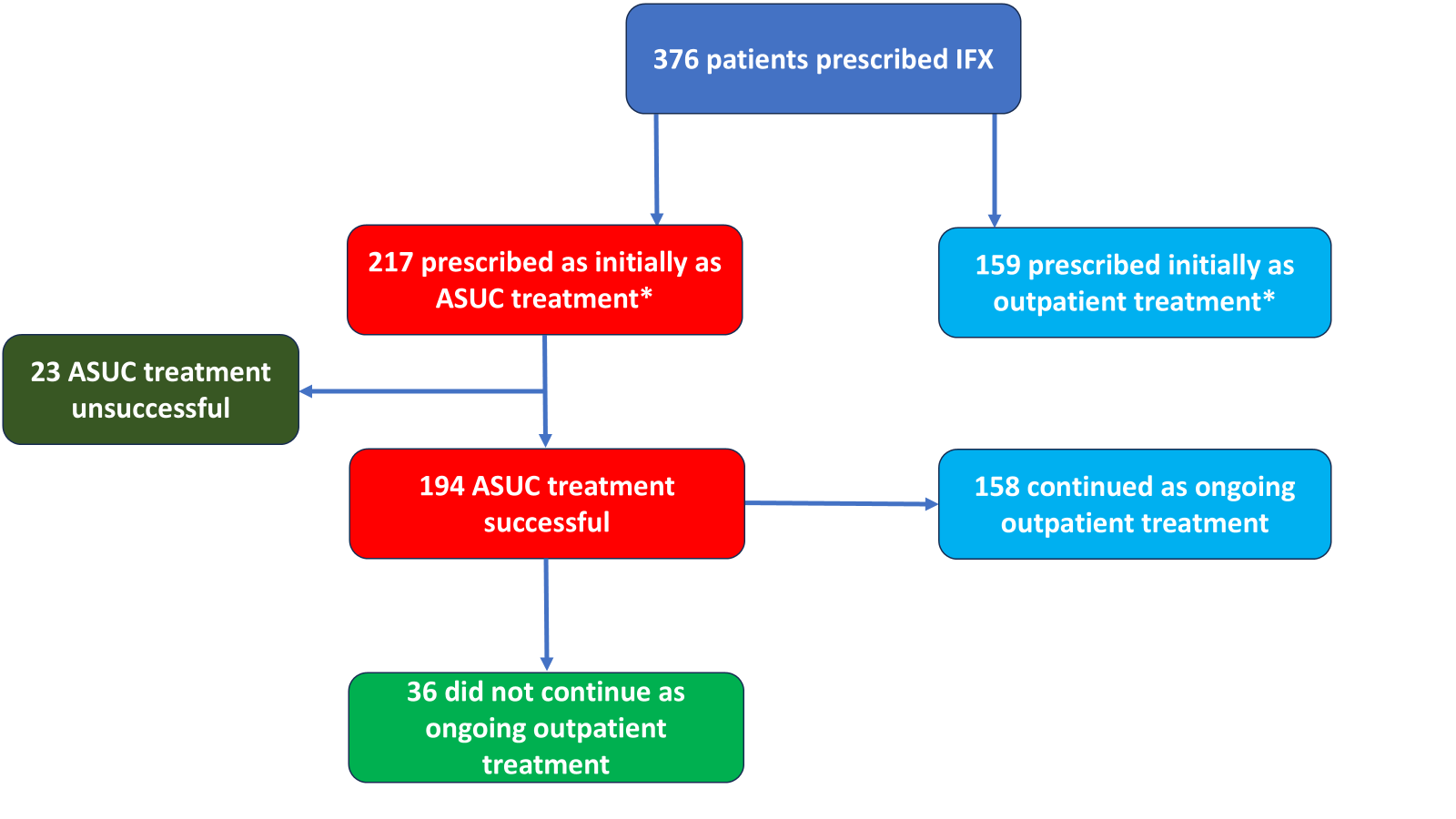
**

**Supplemental Figure 6-** Breakdown of emergency colectomies in the Lothian UC population stratified by time from diagnosis and year. Total number of emergency colectomies= 359. Within 90 days (early)= 114 (32%). Beyond 90 days= 245 (68%). The 2004 data was excluded given the urgency of colectomy was not routinely available.


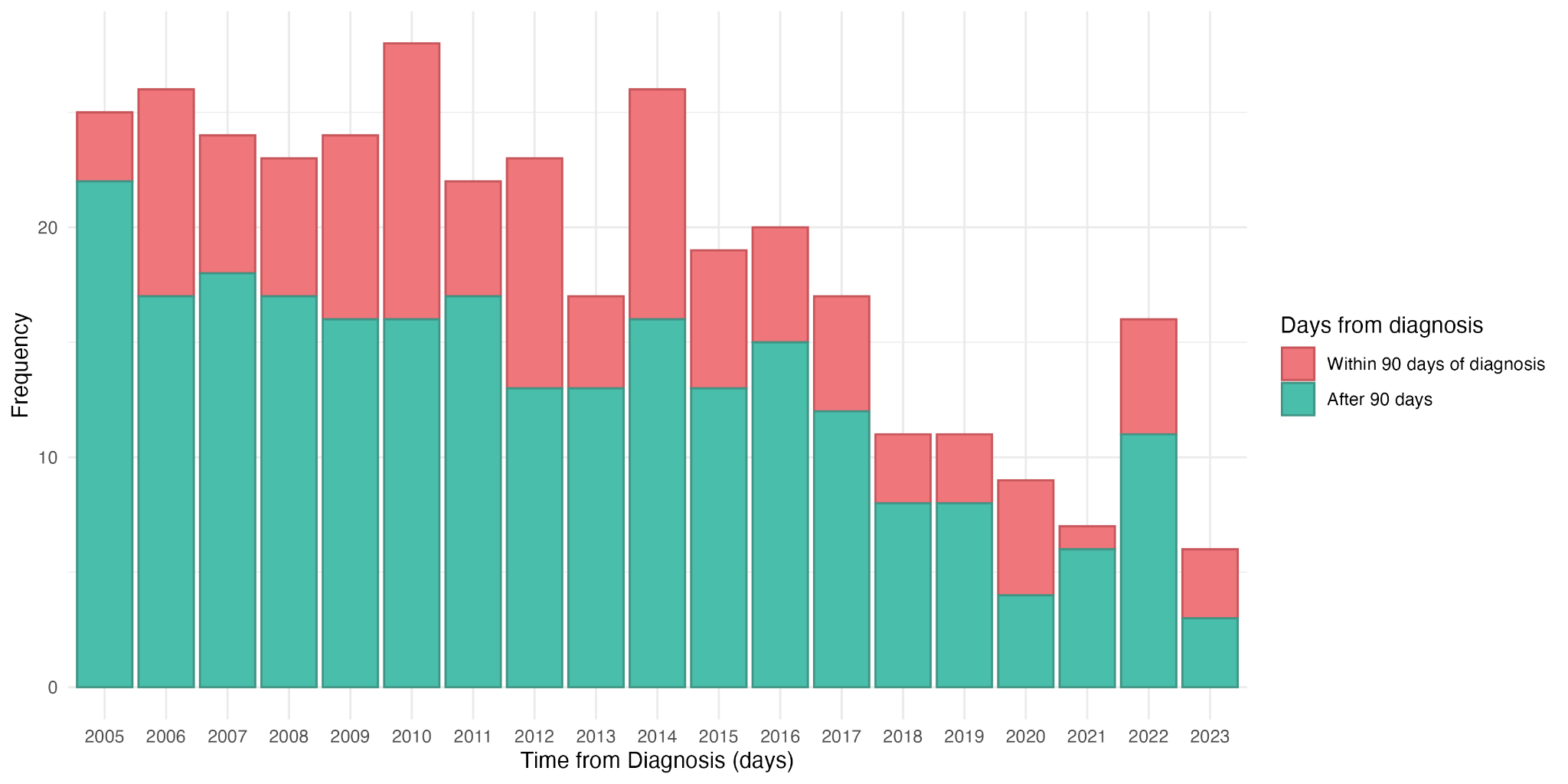


**Supplemental Figure 7-** Breakdown of colectomies in the Lothian UC population according to indication for colectomy over time. Cancer included cancer related concerns such as dysplasia, refractory patients were those who underwent an elective colectomy for medically refractory UC, and ASUC patients were inpatients who had a colectomy for an ulcerative colitis flare. The 2004 data was excluded given inadequate documentation.


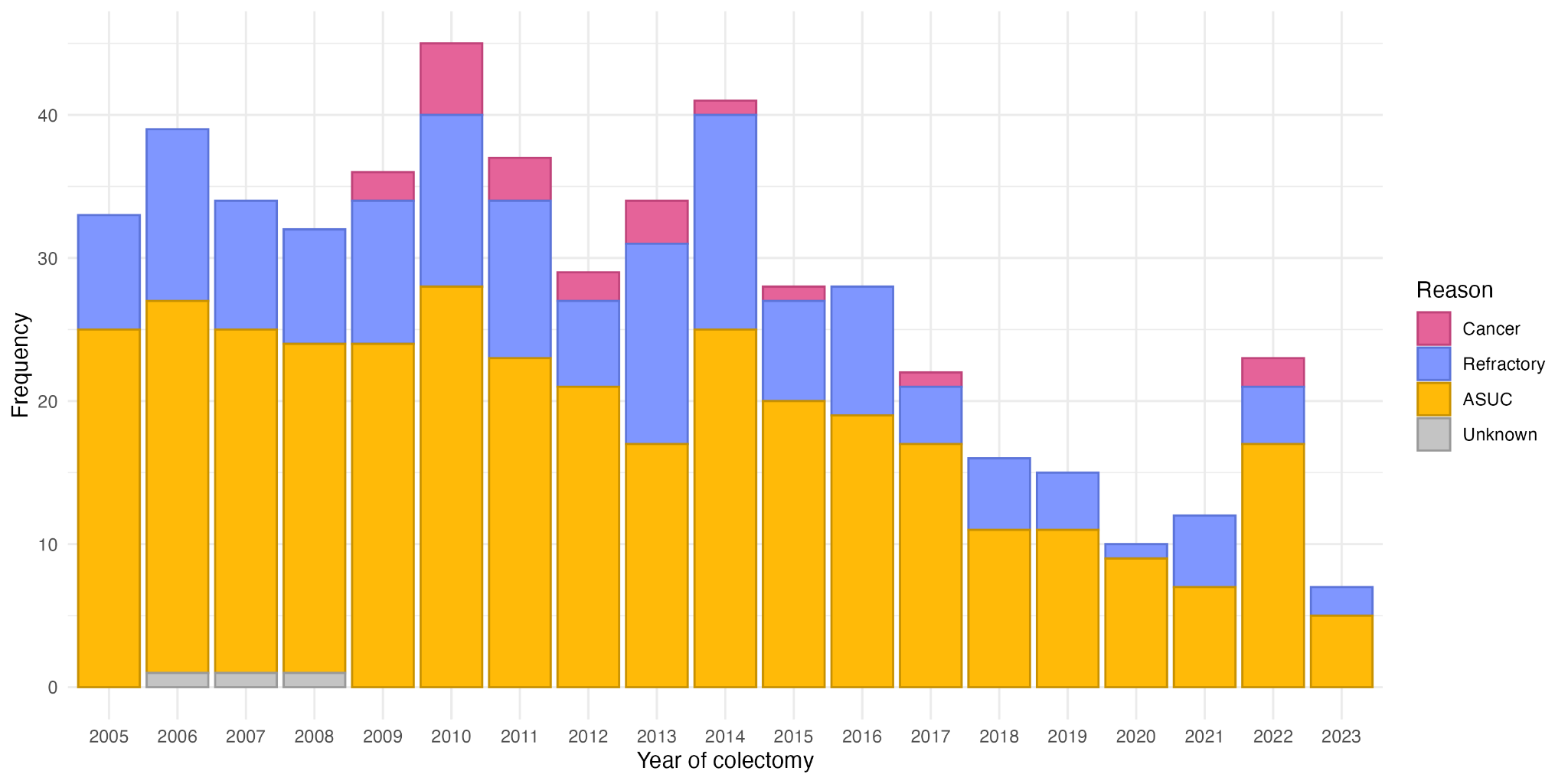


**Supplemental Figure 8-** Complication rates for colectomies in the Lothian UC population over time from 2009-2023. Complications within the first 30 days of the colectomy occurred in 39% (150/382) of this cohort. Data prior to 2009 excluded given inadequate documentation.

**
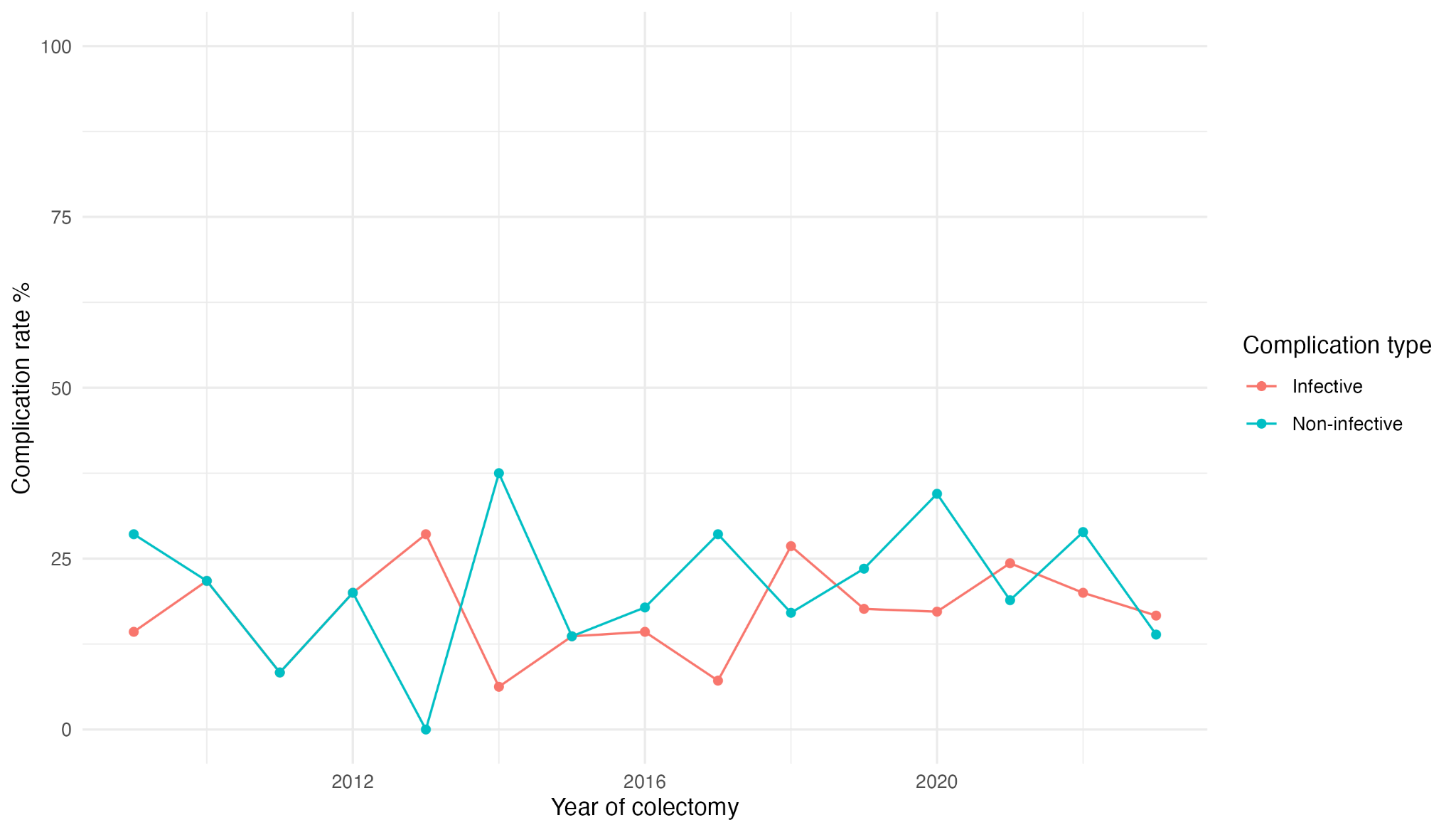
**

**Supplementary material**

**Supplemental Table 1-** Postoperative colectomy complications from 2009-2023 in the Lothian UC population. 2004-2009 data was not included given complication data was not routinely available for this period.

| **Clavien-Dindo classification**^a^ , n (%) | **All patients n=382** |
| --- | --- |
| Grade I | 16 (4%) |
| Grade II | 90 (24%) |
| Grade IIIa | 9 (2%) |
| Grade IIIb | 21 (5%) |
| Grade IV | 8 (2%) |
| Grade V | 6 (2 %) |
| **Complication type,**  n (%) | **All patients n=382** |
| Intra-abdominal infections | 19 (5%) |
| Ileus | 29 (8%) |
| Bowel obstruction | 6 (2%) |
| Rectal Stump blowout | 6 (2%) |
| Bleeding or anaemia requiring blood transfusion | 10 (3%) |
| Surgical complication-other^b^ | 20 (5%)table 2 |
| Wound infection | 17 (4%) |
| Pneumonia | 8 (1%) |
| Other infection | 12 (3%) |
| MACE | 5 (1%) |
| Medical complication-other^c^ | 16 (4%) |

MACE, major adverse cardiovascular event. VTE, venous thromboembolism. ^a^Grade I- Any deviation from the normal postoperative course with only minor therapeutics required such as antiemetics. Grade II- Complication requiring medical treatments such as antibiotics, blood transfusions, and total parenteral nutrition. Grade III- Complication requiring surgical, endoscopic or radiologic intervention (a not under general anaesthesia, b under general anaesthesia). Grade IV- Life threatening complication. Grade V- Death.

^b^Surgical complication other- surgical complications that occurred in <5 people and included anastomotic leak, bowel ischaemia, high stoma output, postoperative vomiting, readmission for gut related symptoms, and abdominal operation within 30 days.

^c^Medical complication other- Medical complications that occurred in <5 people which included acute kidney injury, atrial fibrillation, electrolyte disturbance, cardiovascular or respiratory failure, peptic ulcer, and venous thromboembolism.
